# Sevoflurane posttreatment prevents oxidative and inflammatory injury in ventilator-induced lung injury

**DOI:** 10.1371/journal.pone.0192896

**Published:** 2018-02-22

**Authors:** Julie Wagner, Karl M. Strosing, Sashko G. Spassov, Ziwei Lin, Helen Engelstaedter, Sabine Tacke, Alexander Hoetzel, Simone Faller

**Affiliations:** 1 Department of Anesthesiology and Critical Care Medicine, Medical Center – University of Freiburg, Faculty of Medicine, University of Freiburg, Freiburg, Germany; 2 Department of Veterinary Clinical Sciences, Clinic for Small Animal-Surgery, Justus-Liebig-University Giessen, Giessen, Germany; University of Colorado Denver, UNITED STATES

## Abstract

Mechanical ventilation is a life-saving clinical treatment but it can induce or aggravate lung injury. New therapeutic strategies, aimed at reducing the negative effects of mechanical ventilation such as excessive production of reactive oxygen species, release of pro-inflammatory cytokines, and transmigration as well as activation of neutrophil cells, are needed to improve the clinical outcome of ventilated patients. Though the inhaled anesthetic sevoflurane is known to exert organ-protective effects, little is known about the potential of sevoflurane therapy in ventilator-induced lung injury. This study focused on the effects of delayed sevoflurane application in mechanically ventilated C57BL/6N mice. Lung function, lung injury, oxidative stress, and inflammatory parameters were analyzed and compared between non-ventilated and ventilated groups with or without sevoflurane anesthesia. Mechanical ventilation led to a substantial induction of lung injury, reactive oxygen species production, pro-inflammatory cytokine release, and neutrophil influx. In contrast, sevoflurane posttreatment time dependently reduced histological signs of lung injury. Most interestingly, increased production of reactive oxygen species was clearly inhibited in all sevoflurane posttreatment groups. Likewise, the release of the pro-inflammatory cytokines interleukin-1β and MIP-1β and neutrophil transmigration were completely prevented by sevoflurane independent of the onset of sevoflurane administration. In conclusion, sevoflurane posttreatment time dependently limits lung injury, and oxidative and pro-inflammatory responses are clearly prevented by sevoflurane irrespective of the onset of posttreatment. These findings underline the therapeutic potential of sevoflurane treatment in ventilator-induced lung injury.

## Introduction

Acute lung injury caused by mechanical ventilation alone or in combination with pre-existing pulmonary disease is still a major problem in critical care units [1,2].

The development of lung injury during mechanical ventilation is promoted by a series of traumatic events: Constant cyclic stretching of the lungs leads to tissue disruption and edema formation, thereby stimulating excessive production of reactive oxygen species (ROS). ROS, in turn, can aggravate lung injury by alteration of amino acids and cellular metabolism, peroxidation of cell lipids, or even DNA breakage [3,4]. In addition, mechanical ventilation initiates the development of a profound inflammatory response that is characterized by substantial release of pro-inflammatory cytokines such as interleukin-1β (IL-1β), macrophage inflammatory protein-1β (MIP-1β), or MIP-2 and the transmigration of neutrophils into the bronchoalveolar space [5,6]. Furthermore, activation of these neutrophils enhances the overall inflammatory response, since neutrophils themselves liberate pro-inflammatory cytokines and ROS–thus generating a vicious cycle that may culminate in systemic inflammation and multiorgan failure [7]. Despite improvements in ventilation strategies [8], even low tidal volume ventilation can lead to lung injury, mainly due to regional tidal hyperinflation [9]. Thus, therapeutic options to treat this ventilator-induced lung injury (VILI) are urgently needed.

In this respect, inhaled anesthetics, such as sevoflurane, isoflurane, or desflurane, have come into focus. Their safe use has been proven over decades during inhalational anesthesia. In addition, both isoflurane and sevoflurane might be applied in ´off-label`use on the intensive care units for the purposes of sedation [10].

In addition to their narcotic properties, sevoflurane, isoflurane, and desflurane have been demonstrated to exert cerebral [11], renal [12], and cardiac protection [13] in patients. With respect to animal models, inhaled anesthetics exerted cerebral [14], renal [15], hepatic [15], cardiac [16], and lung protection [17–19].

Because many injurious events take place before treatment is available, from a clinical aspect, it would be interesting to know whether inhaled anesthetics act protectively when applied ex post. In fact, the organ-protective effects of sevoflurane or isoflurane posttreatment were demonstrated for patients receiving a cardiopulmonary bypass [20], as well as for several other experimental injury models [14,15,21–26]. For instance, sevoflurane posttreatment protected rats from cerebral ischemia-reperfusion injury [14,26]. Delayed isoflurane application prevented renal or hepatic injury in a cecal ligation and puncture model in mice [15]. Likewise, delayed sevoflurane application resulted in substantial protective effects in several models of cardiac ischemia-reperfusion injury in rats [21,22,24,25] and mice [23].

With regard to ventilator-induced lung injury, we and others demonstrated lung protective effects of sevoflurane and isoflurane, when inhalation was commenced at the onset of mechanical ventilation [17–19]. Even if two injury models in dogs using sevoflurane posttreatment showed lung protection after cardiopulmonary bypass [27] or in oleic acid-induced lung damage [28], it has never been studied, whether postponed application of sevoflurane effectively protects against VILI. That knowledge would be most important for the future of ventilated patients developing VILI on the critical care unit.

The aim of the current study was therefore to further analyze the effects of sevoflurane posttreatment on organ injury, oxidative burst, and inflammatory stress in the lung during mechanical ventilation. Here, we provide evidence that inhaled sevoflurane profoundly limits oxidative and inflammatory responses, as well as organ injury, at a time point when VILI has already been established.

## Materials and methods

### Ethics committee approval

The care of animal and licensing guidelines under which the study was performed were in accordance with the ARRIVE (Animals in Research: Reporting In Vivo Experiments) statement and the journals`requirements. Animal experiments were performed in accordance with the guidelines of the local animal care commission (University of Freiburg, Freiburg, Germany). The study was approved by the local government, which had been advised by an ethics committee (Regierungspräsidium Freiburg, Freiburg, Germany, permission No. G-07/25). All surgery was performed under deep ketamine/acepromazine or sevoflurane anesthesia, and all efforts were made to minimize suffering.

### Experimental groups

**Fig 1:**
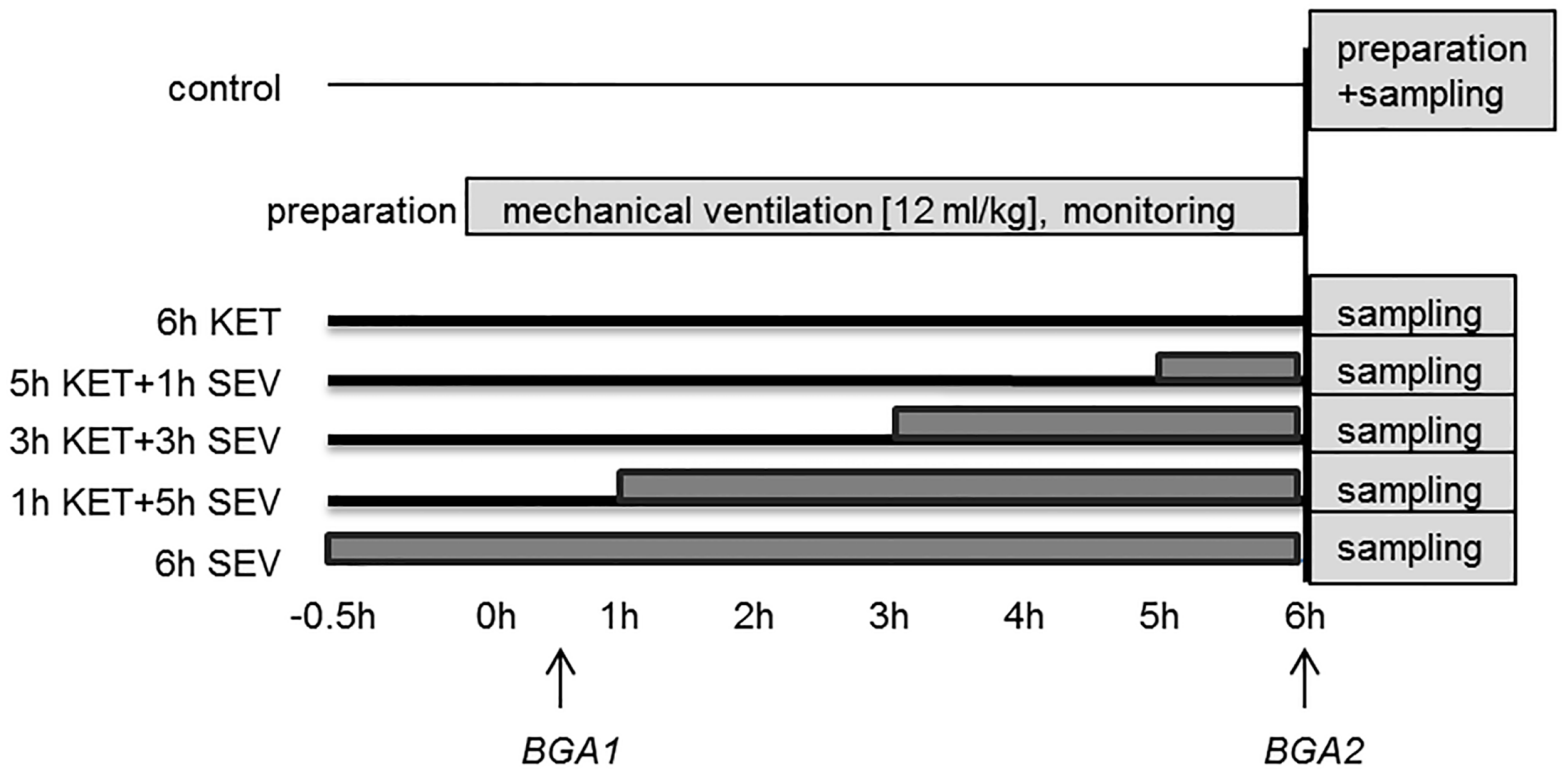
Study timeline. Mice were either non-ventilated (control), or they were mechanically ventilated with 12 ml/kg for 6 h either under ketamine + acepromazine (6h KET) or sevoflurane (6h SEV) anesthesia or a combination of both: KET for 5, 3, or 1 h, followed by SEV for another 1, 3, or 5 h as indicated (5h KET + 1h SEV, 3h KET + 3h SEV, 1h KET + 5h SEV).

Mice were randomized into 6 experimental groups (n = 7/group; Fig 1). Group 1 served as a control group with mice spontaneously breathing air for 6 hours (control). All other groups were subjected to mechanical ventilation for 6 h. Group 2: mice were anesthetized with ketamine + acepromazine for 6 h (6h KET). Group 3: mice were anesthetized with ketamine + acepromazine for 5 h followed by sevoflurane for 1 h (5h KET+1h SEV). Group 4: mice were anesthetized with ketamine + acepromazine for 3 h followed by sevoflurane for another 3 h (3h KET+3h SEV). Group 5: mice were anesthetized with ketamine + acepromazine for 1 h followed by sevoflurane for another 5 h (1h KET+5h SEV). Group 6: animals were anesthetized with sevoflurane for 6 h (6h SEV).

### Experimental setup

Male C57BL/6N mice (weighing 24 g ± 1 g) were obtained from Charles River Laboratories (Sulzfeld, Germany). Until the onset of the experiment, mice were housed in standard cages at ambient temperatures of 22°C (± 1°C), 12 h dark/ 12 h light cycle, and had free access to food and water. Mice were then anesthetized intraperioneally (*i*.*p*.) with 90 mg/kg ketamine (Ketanest, 50 mg/ml, Inresa Arzneimittel GmbH, Freiburg, Germany) and 0.9 mg/kg acepromazine (Vetranquil 1%, Ceva Tiergesundheit GmbH, Düsseldorf, Germany) or inhaled sevoflurane (3.28 ± 0.34%Vol; MAC 1.0; AbbVie Deutschland GmbH & Co. KG, Wiesbaden, Germany) [23]. As described earlier [18], an arterial line and a tracheal tube were inserted. In the case of mechanical ventilation, mice were then connected to a rodent ventilator (Voltekenterprises, Toronto, ON, Canada), set to a tidal volume of 12 ml/kg, frequency of 80–90 breaths/minute, and positive end-expiratory pressure (PEEP) of 2 cm H_2_O for 6 h. Alveolar recruitment maneuvers were performed every 60 minutes (5 seconds of inspiratory pressure hold at 30 cm H_2_O). Muscle relaxation was induced directly after the onset of mechanical ventilation and during the experiment by injection of pancuronium (2 mg/kg, *i*.*p*.). Peak pressure (Ppeak), plateau pressure (Pplateau), and mean arterial pressure (MAP) were monitored throughout the experiment and recorded every 30 min. Static compliance was calculated for each time point from the recorded Pplateau values divided by the individual tidal volumes. Blood gas analyses were performed at the beginning (after 30 min of ventilation) and at the end of the ventilation period (6 h, Fig 1), and pH, arterial oxygen partial pressure (Pa_O2_), and arterial carbon dioxide partial pressure (Pa_CO2_) were measured. General anesthesia was either maintained by continuous *i*.*p*. administration of ketamine and acepromazine as needed or sevoflurane inhalation. At the end of each experiment, the mice were sacrificed by an overdosed injection of ketamine and acepromazine.

### Bronchoalveolar lavage and lung preparation

Bronchoalveolar lavage (BAL) fluid was gained by flushing the right lung lobes with 0.8 ml PBS. 0.6 ml ± 0.06 ml were recovered without significant differences between groups and were stored at -80 °C until further use. For ROS detection in subsequent experiments, a part of the right lung lobe was additionally embedded into optimal cutting temperature compound (Tissue Tek, Sakura Finetek Germany GmbH, Staufen, Germany) and again stored at -80 °C. The left lungs were inflated with 2% paraformaldehyde under a constant pressure of 20 cm H_2_O. Subsequently, they were extracted, incubated in paraformaldehyde for another 2 h, followed by storage in 30% sucrose at 4 °C overnight. Prior to preservation at -80 °C, the left lungs were embedded in optimal cutting temperature compound and slowly frozen on liquid nitrogen [29].

### Histological analysis

6 μm cryosections from all areas of the fixed left lung were stained with hematoxylin and eosin and analyzed (magnification 200x). At least five photos per lung were taken and alveolar wall thickness was measured within each photo in five predefined fields with AxioVision 4.8 software (Carl Zeiss, Jena, Germany). To assess the degree of lung damage by VILI score, measured alveolar wall thickness, infiltrating cells per field and a hemorrhage score per photo were additionally rated from 0 (no lung damage) to 4 (maximal lung damage), and summed up per lung [29]. All histological analyses were performed in a blinded fashion.

### Detection of reactive oxygen species (ROS)

6 μm cryosections from all areas of the right lung lobe were stained with 20 μM dihydroethidium (DHE, Life Technologies GmbH, Darmstadt, Germany), covered, and incubated at 37°C for 30 minutes in a dark humidified chamber. Red fluorescence was detected with a confocal laser scanning microscope (magnification 200x, LSM 510 META NLO, Carl Zeiss). 3–4 images/lung were taken and fluorescence intensity per cell were analyzed with ImageJ software (NIH, Bethesda, USA).

### Neutrophil analysis

BAL fluid cells were separated by centrifugation and stained with Diff-Quik^®^ (Siemens Healthcare Diagnostics, Eschborn, Germany). In total, 200 to 300 living cells per sample were counted under the microscope and the fraction of neutrophils was determined.

### Cytokine measurements

BAL supernatant was analyzed using Pierce^®^ BCA Protein Assay (Thermo Fisher Scientific, Darmstadt, Germany). Further BAL analysis included interleukin-1β (IL-1β), macrophage inflammatory protein-1β (MIP-1β) and MIP-2 ELISA kits (R&D Systems GmbH, Wiesbaden, Germany) that were applied according to the manufacturers’ instructions. The amount of IL-1β, MIP-1β, and MIP-2 was normalized to total protein concentrations in BAL supernatant and compared among groups.

### Statistical analysis

All animal experiments were performed with n = 7 mice per group. Power calculations were performed prior to the study in order to define group sizes. The data in tables and graphs represent means ± standard deviation (SD). Graphs were created with SigmaPlot 11.0 (Systat Software Inc., Erkrath, Germany). Data were further analyzed for normal variation prior to one way analysis of variance (ANOVA) followed by the Tukey`s post hoc test, comparing the mean of each column with the mean of each other column, or t-Test as indicated. *P*<0.05 was considered significant. All calculations were performed with GraphPad Prism 7.01 (GraphPad Software, Inc., La Jolla, CA USA).

## Results

### Effect of sevoflurane posttreatment on physiological parameters

In order to study the effect of sevoflurane posttreatment during 6 h of mechanical ventilation, mice were either subjected to ketamine or sevoflurane anesthesia alone, or initial ketamine anesthesia for 5, 3, or 1 h was followed by anesthesia with sevoflurane for another 1, 3, or 5 h, respectively (study timeline, Fig 1).

In the first set of analyses, we sought to investigate whether sevoflurane posttreatment would interfere with physiological parameters such as arterial blood gases or lung function.

With respect to arterial blood gas analysis, our readings for Pa_O2_ and Pa_CO2_ showed no differences between groups, except that the pH was reduced after 6 h sevoflurane anesthesia alone, compared to sevoflurane treatment in the last hour of ventilation (6h SEV vs. 5h KET+1h SEV; Table 1). Blood pressure measurements revealed a reduced mean arterial pressure when sevoflurane anesthesia was initiated after 1 h ketamine anesthesia (1h KET+5h SEV) as compared to the 6 h ketamine anesthesia as well as compared to sevoflurane anesthesia in the last hour of ventilation (6h KET and 5h KET+1h SEV; Table 1).

**Table 1:** Physiological parameters.

	6h KET	5h KET +1h SEV	3h KET +3h SEV	1h KET +5h SEV	6h SEV	*P*
	**BGA parameters after 6h**					
**Pa**_**O2**_ **[mmHg]**	81.8 (± 9.1)	71.9 (± 27.8)	75.4 (± 8.9)	84.2 (± 12.5)	71.2 (± 11.1)	*ns*
**Pa**_**CO2**_ **[mmHg]**	43.8 (± 10.7)	31.8 (± 4.7)	36.1 (± 9.5)	41.8 (± 13.4)	44.7 (± 8.2)	*ns*
**pH**	7.311 (± 0.074)	7.395 (± 0.028)	7.362 (± 0.077)	7.343 (± 0.102)	7.285^+^ (± 0.050)	*< 0*.*05*
	**6h average**					
**mP**_**Peak**_ **[mmHg]**	10.3 (± 0.8)	10.1 (± 0.3)	10.3 (± 0.6)	10.1 (± 0.7)	10.6 (± 0.7)	*ns*
**mP**_**Plateau**_**[mmHg]**	7,9 (± 0.7)	7,7 (± 0.2)	7,9 (± 0.6)	7,8 (± 0.7)	7,5 (± 0.8)	*ns*
**MAP** **[mm Hg]**	67 (± 11)	65 (± 5)	58 (± 3)	53^#^^+^ (± 3)	58 (± 5)	*< 0*.*05*

Mice were mechanically ventilated with 12 ml/kg for 6 h either under ketamine + acepromazine (6h KET) or sevoflurane (6h SEV) anesthesia or a combination of both: KET for 5, 3, or 1 h, followed by SEV for another 1, 3, or 5 h as indicated (5h KET + 1h SEV, 3h KET + 3h SEV, 1h KET + 5h SEV). pH, arterial oxygen partial pressure (Pa_O2_), and arterial carbon dioxide partial pressure (Pa_CO2_) were measured at the end of the experiment. Peak pressure (P_Peak_), plateau pressure (P_Plateau_), and mean arterial pressure (MAP) were monitored throughout ventilation and values were recorded every 30 min. Data represent means ± SD for n = 5 (6h KET; 6h SEV in Pa_O2_), n = 6 (1h KET + 5h SEV; 3h KET + 3h SEV in Pa_O2_), or n = 7/group. ANOVA (Tukey`s post hoc test),

^#^*P*<0.05 vs. 6h KET group;

^+^*P*<0.05 vs. 5h KET + 1h SEV vent group.

Ventilatory readings, such as mean peak and plateau pressure, as well as mean static compliance (mC_stat_), as parameters for mechanical lung function, were not different among groups (Tables 1 and 2). Furthermore, mean static compliance did not vary before or during sevoflurane anesthesia between groups. Only within one experimental group, starting with 5 h ketamine anesthesia, followed by 1 h of sevoflurane anesthesia (5h KET+1h SEV), the mean static compliance within the time frame of ketamine anesthesia showed to be higher as compared to the subsequent sevoflurane anesthesia (Table 2).

**Table 2:** Static compliance before and after sevoflurane application.

	6h KET	5h KET +1h SEV	3h KET +3h SEV	1h KET +5h SEV	6h SEV	*P*
	**6h average**					
**mC**_**stat**_ **[μl/cmH**_**2**_**O]**	39 (± 3)	40 (± 2)	41 (± 4)	38 (± 3)	37 (± 5)	*ns*
	**before SEV application**					
**mC**_**stat**_ **[μl/cmH**_**2**_**O]**	39 (± 3)	41 (± 2)	42 (± 3)	39 (± 2)		*ns*
	**during SEV application**					
**mC**_**stat**_ **[μl/cmH**_**2**_**O]**		36 (± 2)	40 (± 4)	38 (± 3)	37 (± 5)	*ns*
***P (before vs after)***		*< 0*,*0001*	*ns*	*ns*		

Mice were mechanically ventilated with 12 ml/kg for 6 h either under ketamine + acepromazine (6h KET) or sevoflurane (6h SEV) anesthesia or a combination of both: KET for 5, 3, or 1 h, followed by SEV for another 1, 3, or 5 h as indicated (5h KET + 1h SEV, 3h KET + 3h SEV, 1h KET + 5h SEV). Mean static compliance (mC_stat_) was monitored throughout ventilation and depicted as mean before and during SEV application. Data represent means ± SD for n = 6 (3h KET + 3h SEV; 6h SEV) or n = 7/group. ANOVA (Tukey`s post hoc test) for comparisons between all groups, t-Test for intergroup comparisons (before and after SEV treatment).

These results suggest that sevoflurane anesthesia did not interfere with lung function.

### Effect of sevoflurane posttreatment on ventilator-induced lung injury

**Fig 2:**
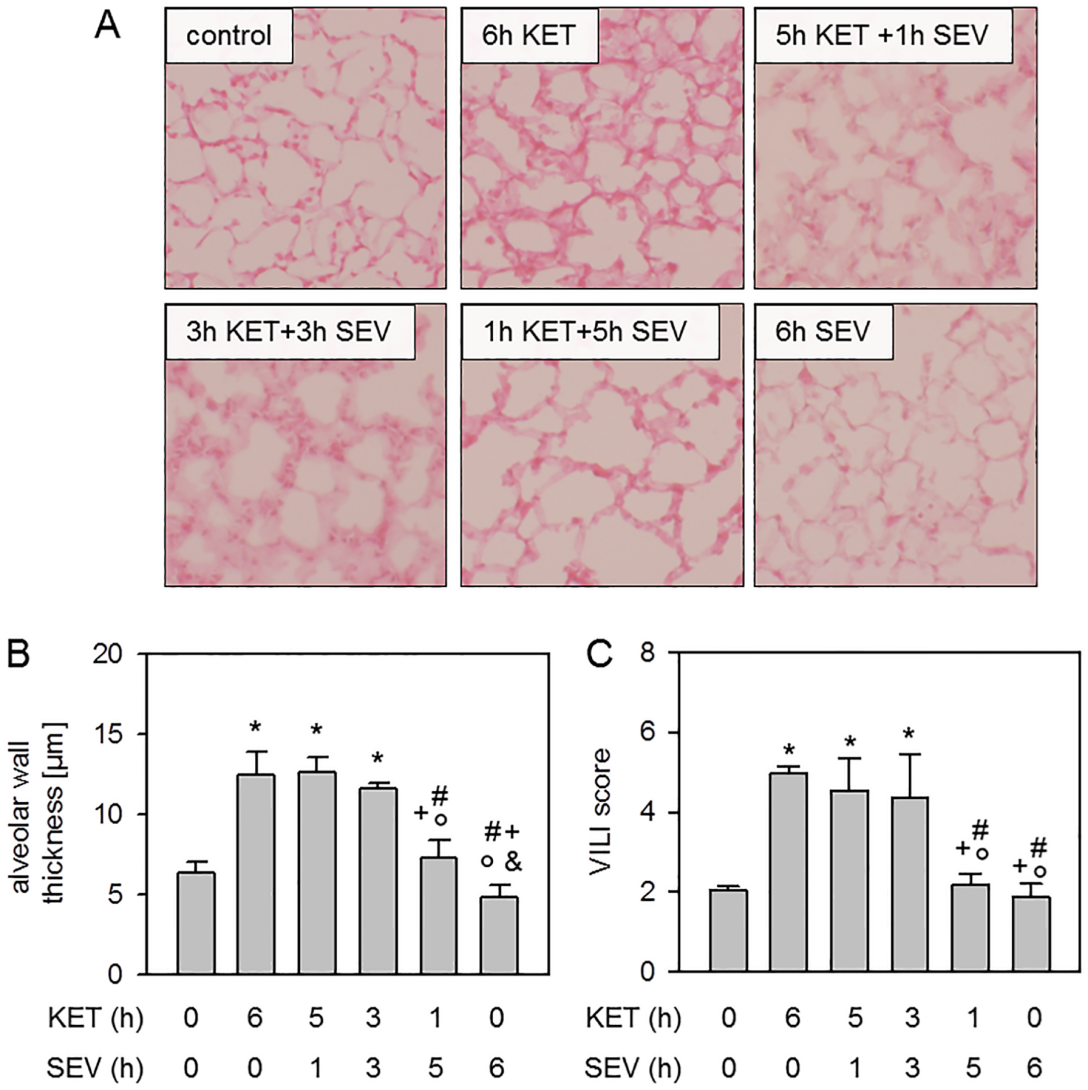
Effect of sevoflurane posttreatment on ventilator-induced lung injury. Mice were either non-ventilated (control), or they were mechanically ventilated with 12 ml/kg for 6 h either under ketamine + acepromazine (6h KET) or sevoflurane (6h SEV) anesthesia or a combination of both: KET for 5, 3, or 1 h, followed by SEV for another 1, 3, or 5 h as indicated (5h KET + 1h SEV, 3h KET + 3h SEV, 1h KET + 5h SEV). Lung tissue sections were stained with hematoxylin and eosin. Representative pictures are shown for each experimental group as indicated (200x, A). High power fields were randomly assigned to measure alveolar wall thickness (B), and to calculate a ventilator-induced lung injury (VILI) score (C). Data represent means ± SD for n = 7/group. ANOVA (Tukey`s post hoc test), *P<0.05 vs. control group; ^#^*P*<0.05 vs. 6h KET group; ^+^*P*<0.05 vs. 5h KET + 1h SEV group; °*P*<0.05 vs. 3h KET + 3h SEV group; ^&^*P*<0.05 vs. 1h KET + 5h SEV group.

We next hypothesized that sevoflurane posttreatment might improve the degree of lung injury within 6 h of injurious mechanical ventilation. In comparison to non-ventilated controls, 6 h of ketamine anesthesia alone (6h KET) as well as inhalation of sevoflurane for the last hour (5h KET+1h SEV) or the last 3 hours (3h KET+3h SEV) resulted in histopathological signs of lung damage (Fig 2A). Quantification of these findings by measurement of alveolar wall thicknesses (Fig 2B) and applying the VILI score (Fig 2C) confirmed these findings and demonstrated increased lung injury when sevoflurane application was postponed up to 3 h. In contrast, lung damage was clearly reduced to control levels in mice receiving sevoflurane posttreatment for 5 h (1h KET+5h SEV) or sevoflurane anesthesia for the whole ventilation period of 6 h without additional ketamine anesthesia (6h SEV; Fig 2A to 2C). These results indicate that morphological lung injury may be prevented by starting sevoflurane inhalation as early as possible.

### Effect of sevoflurane posttreatment on oxidative stress

**Fig 3:**
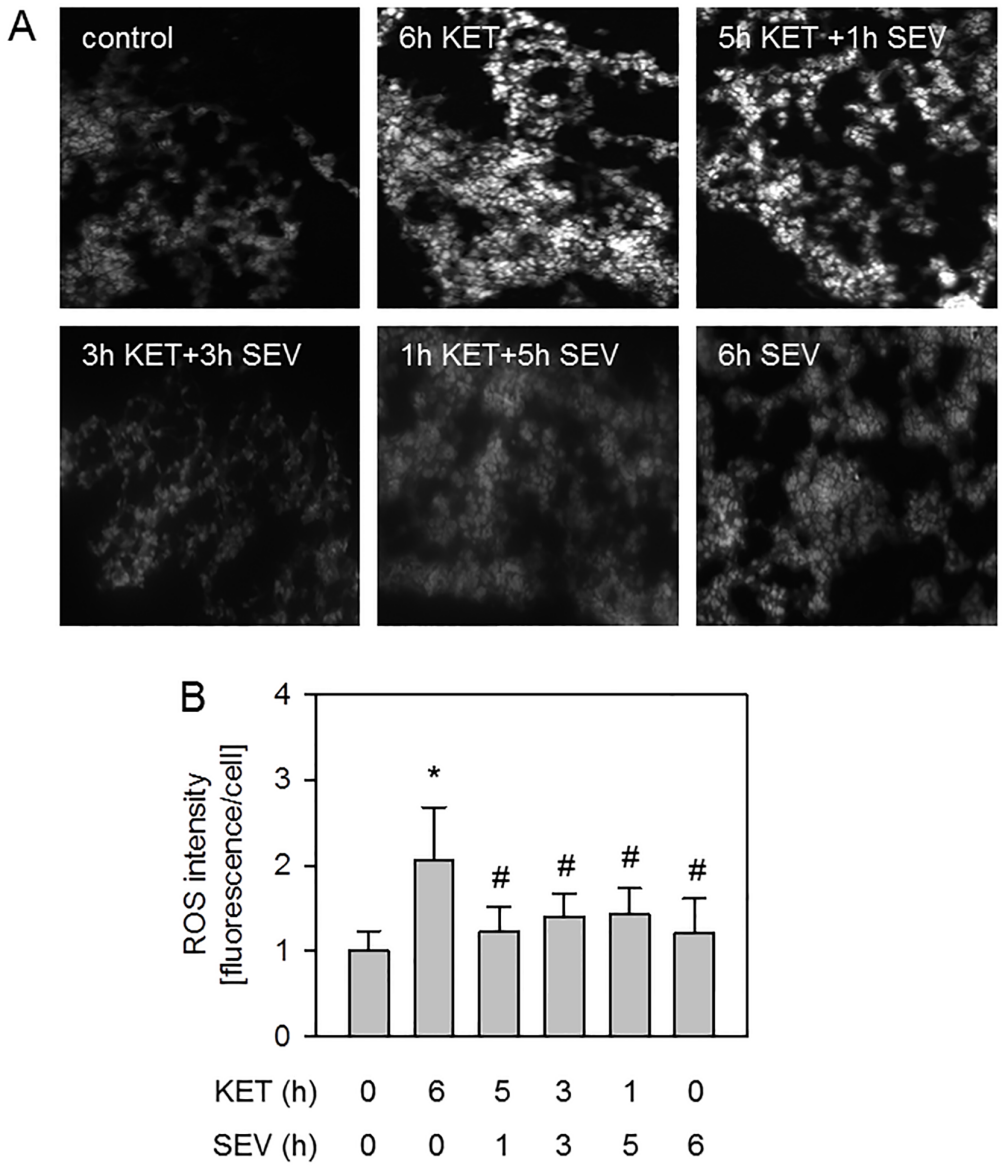
Effect of sevoflurane posttreatment on oxidative stress. Mice were either non-ventilated (control), or they were mechanically ventilated with 12 ml/kg for 6 h either under ketamine + acepromazine (6h KET) or sevoflurane (6h SEV) anesthesia or a combination of both: KET for 5, 3, or 1 h, followed by SEV for another 1, 3, or 5 h as indicated (5h KET + 1h SEV, 3h KET + 3h SEV, 1h KET + 5h SEV). Lung tissue sections were stained by DHE (A). Representative pictures are shown for each experimental group as indicated (200x, A). ROS fluorescence intensity per cell was measured and expressed as fold induction compared to control group (B). Data represent means ± SD for n = 4 (5h KET + 1h SEV) or n = 7/group. ANOVA (Tukey`s post hoc test), *P<0.05 vs. control group; ^#^*P*<0.05 vs. 6h KET group.

Reactive oxygen species (ROS) play a central role in the development of VILI [3]. We recently showed that sevoflurane anesthesia applied during the entire ventilation period significantly reduced ROS formation in VILI [18] and we sought to examine whether sevoflurane posttreatment would also be sufficient to prevent ROS formation. DHE staining of lung cryosections and its quantification demonstrated that air ventilation for 6 h under ketamine anesthesia alone led to increased ROS production compared to non-ventilated controls (Fig 3A and 3B). In contrast, inhalation of sevoflurane time independently abolished ROS formation despite mechanical ventilation and was comparable to controls (Fig 3A and 3B). These results suggest that oxidative stress is inhibited due to sevoflurane anesthesia, irrespective of the onset of posttreatment.

### Effect of sevoflurane posttreatment on lung inflammation

**Fig 4:**
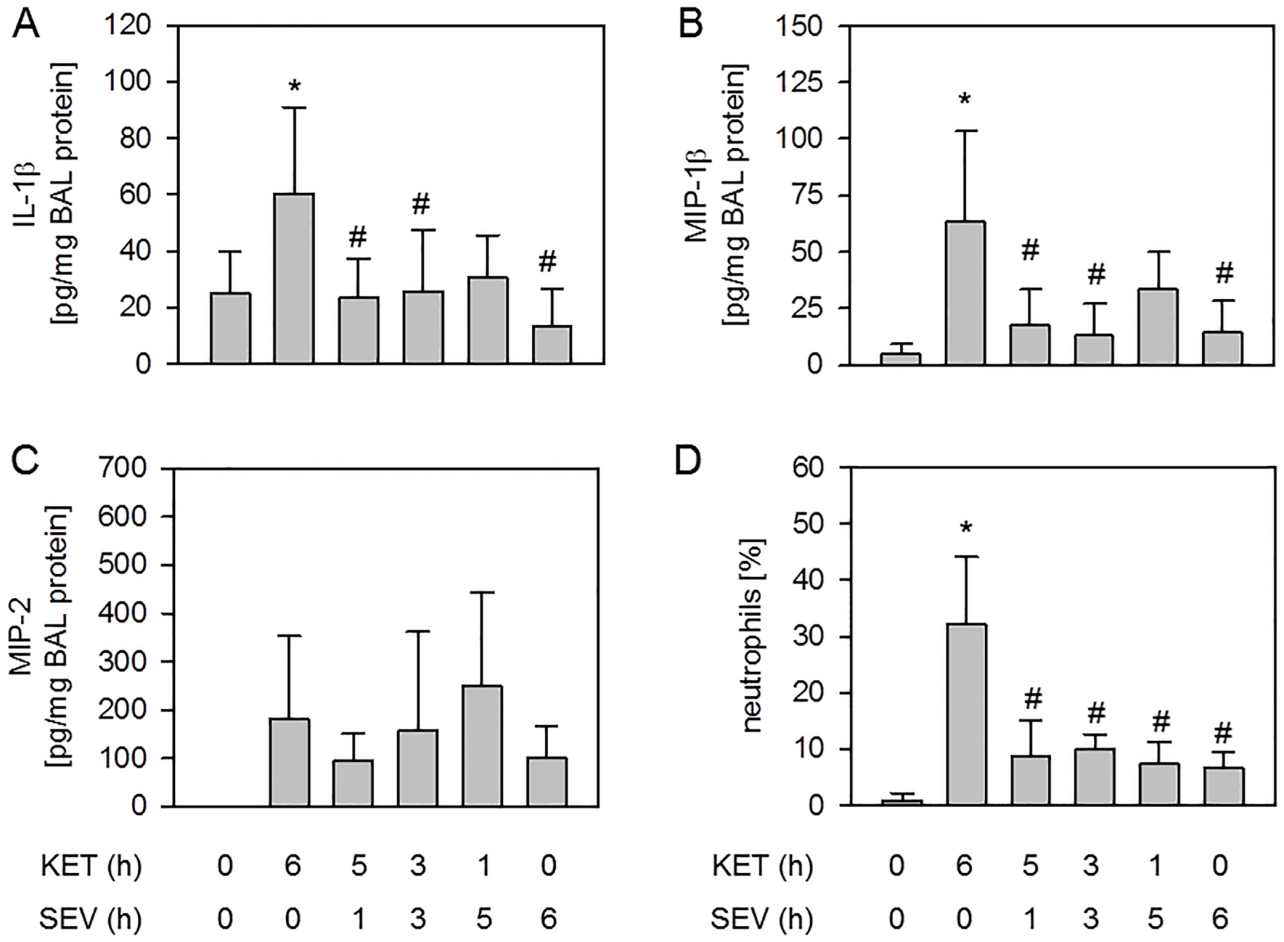
Effect of sevoflurane posttreatment on lung inflammation. Mice were either non-ventilated (control), or they were mechanically ventilated with 12 ml/kg for 6 h either under ketamine + acepromazine (6h KET) or sevoflurane (6h SEV) anesthesia or a combination of both: KET for 5, 3, or 1 h, followed by SEV for another 1, 3, or 5 h as indicated (5h KET + 1h SEV, 3h KET + 3h SEV, 1h KET + 5h SEV). The content of IL-1β (A), MIP-1β (B), and MIP-2 (C) was determined by ELISA and the relative amount of neutrophils (D) was determined by cytospin analysis in the bronchoalveolar lavage fluid. Data represent means ± SD for n = 5-7/group (A+C) and n = 7/group (D). ANOVA (Tukey`s post hoc test), *P<0.05 vs. control group; ^#^*P*<0.05 vs. 6h KET group.

Besides the production of excessive ROS, lung injury due to mechanical ventilation is promoted by an inflammatory response, *i*.*e*. the release of pro-inflammatory cytokines and the transmigration of neutrophils into the alveolar compartment [30]. We have demonstrated previously that inhaled anesthetics may reduce pro-inflammatory signaling [17,18] and aimed in this study to determine if posttreatment with the inhaled anesthetic sevoflurane would also be capable of limiting inflammation. Compared to non-ventilated controls, the pro-inflammatory cytokines IL-1β (Fig 4A), MIP-1β (Fig 4B), and MIP-2 (Fig 4C) were increased in response to mechanical ventilation under 6 h of ketamine anesthesia. While MIP-2 was unaffected by sevoflurane treatment (Fig 4C), both IL-1β (Fig 4A) and MIP-1β (Fig 4B) cytokine release was reduced to control levels independent of the time point of sevoflurane application. Likewise, 6 h ventilation under ketamine anesthesia alone led to a vast influx of neutrophils into the bronchoalveolar space compared to non-ventilated controls, as shown by the relative amount of neutrophil cells in the BAL (Fig 4D). Posttreatment with sevoflurane and 6 h sevoflurane anesthesia alone prevented neutrophil sequestration back to control levels, irrespective of the onset or duration of sevoflurane inhalation (Fig 4D). Taken together, the current data propose that even a late onset of sevoflurane posttreatment is sufficient to limit the pro-inflammatory response in VILI.

## Discussion

In the current study, we analyzed the organ-protective potential of the inhaled anesthetic sevoflurane in a mouse model of ventilator-induced lung injury (VILI). We showed that sevoflurane anesthesia exerts lung protective effects, i.e. reducing morphological injury, oxidative stress, and the inflammatory response. Postponed treatment with sevoflurane prevented morphological lung injury in a time-dependent fashion–the earlier sevoflurane was applied the more lung protection was achieved. Most interestingly, oxidative stress and inflammation were prevented irrespective of the onset and duration of sevoflurane posttreatment.

These results appear to be of clinical relevance, since mechanical ventilation even without underlying pulmonary disease and under low tidal volume ventilation can cause lung injury [1,2,9], still leading to unacceptably high rates of mortality and morbidity in the intensive care unit [7]. The presented data supply first evidence that sevoflurane posttreatment may provide a therapeutic option to reduce VILI.

### Sevoflurane posttreatment and lung function

Mechanical ventilation even with low tidal volumes can induce or aggravate lung injury in critical care patients [1,2] and in animal models [17,18,29,31,32]. Although low tidal volume ventilation (6–8 ml/kg) is favored in clinical practice [8], we decided to ventilate mice with a moderate tidal volume of 12 ml/kg [17,18,29,31]. This regime mimics regions of overinflation in patients with nonaerated lung compartments even during low tidal volume ventilation [9].

The model chosen intended to evoke only moderate lung injury. In that purpose, mechanical ventilation neither deteriorated lung function with respect to Pa_O2_, Pa_CO2_, and pH, nor substantially affected lung mechanical function as expressed by inspiratory peak and plateau pressure and mean lung compliance. In addition, these measurements and their comparability among groups provide evidence that sevoflurane does not significantly alter lung mechanics. Although the mean arterial pressure of all the ventilated mice appear to be lower compared to earlier studies [18], all other parameters analyzed lie within the normal range for laboratory C57BL/6N mice [33,34]. Our results confirm that the chosen ventilator regime avoided undesirable side effects of mechanical ventilation, such as hyperinflation of the lung, hyper- or hypoxia, alkalosis or acidosis, etc. that might have interfered with interpretation of the data set.

### Sevoflurane posttreatment and lung injury

In the current study, we characterized lung injury by histological analysis of alveolar wall thickening and applying a VILI score. Both values reveal that a moderate lung injury evolved upon mechanical ventilation with 12 ml/kg for 6 h. In this respect, lung edema formation was detectable by thickened alveolar walls. However, we did not additionally observe increased albumin in the bronchoalveolar lavage (control: 373 pg/ml +/- 116 pg/ml; 6h KET: 441 pg/ml +/- 89 pg/ml; 5hKET + 1hSEV: 309 pg/ml +/- 59 pg/ml; 3hKET + 3hSEV: 316 pg/ml +/- 59 pg/ml; 1hKET + 5h SEV: 368 pg/ml +/- 95 pg/ml; 6h SEV: 416 pg/ml +/- 115 pg/ml; ANOVA: no significant differences between groups), suggesting that our ventilatory setup promoted only a mild form of lung edema. This appears to be reasonable, since we did not observe impaired oxygenation or deteriorated mechanical lung function, that both would have contributed to a severe form of edema in acute lung injury [35]. We have previously shown that in contrast to air ventilation under *i*.*p*. ketamine anesthesia, sevoflurane application throughout the experiment clearly inhibits the development of lung tissue injury [18]. In our current investigation we observed that sevoflurane inhalation failed to prevent lung injury when given at the end of the ventilation period for 1 or 3 h (following 5 or 3 h of KET anesthesia, respectively). In these cases, lung edema formation and VILI score were as high as after 6 h of ketamine anesthesia alone. Similar observations have been made in ischemia-reperfusion models [36,37]. Sevoflurane inhalation for 3 h during reperfusion was not sufficient to reduce histological lung damage in rats [37] or pigs [36]. In contrast, early onset of sevoflurane posttreatment in our model (1 h KET followed by 5 h SEV treatment) resulted in a profound decrease in histopathological signs of lung injury and was comparable to non-ventilated controls as well as to mice inhaling sevoflurane throughout 6 h of ventilation. These findings suggest that the promotion of edema formation due to mechanical ventilation (1) starts early during ventilation, and that (2) sevoflurane cannot initiate reversal of or remodeling processes of lung tissue damage in a posttreatment setting. The latter might be limited to our time frame of observation. It may be that reversing edema formation by sevoflurane needs more time to be displayed histologically. Therefore, we further investigated the role of promotors of lung injury.

### Sevoflurane posttreatment and oxidative stress

In this respect, the excessive formation of reactive oxygen species (ROS), due to shear stress during mechanical ventilation has been closely linked to the development of organ-damage in acute and ventilator-induced lung injury [3]. Results from our current and previous work show that 6 h sevoflurane treatment substantially inhibited ROS formation in lung tissue during mechanical ventilation [18]. The effects of sevoflurane posttreatment on ROS production have not yet been studied in VILI models. Attempts have been made to investigate the so called postconditioning effects of sevoflurane on ROS in cardiac ischemia-reperfusion models [21,38]. ROS intensity was clearly decreased in rat hearts, when sevoflurane was supplemented for 15 min at the onset of reperfusion [21,38]. In our model, even sevoflurane application in the last hour of mechanical ventilation was sufficient to significantly reduce ROS formation. This is also true for 3 h and 5 h sevoflurane posttreatment that completely prevented ROS production. How sevoflurane posttreatment modulates ROS formation during VILI still remains elusive and needs to be addressed in future studies. Nonetheless, several molecular signaling pathways affected by sevoflurane are conceivable: We have shown recently that Akt phosphorylation is both involved in protection from VILI [17,39] and is associated with the reduction of ROS formation [39]. Moreover, Zhang *et al*. showed in a model of cardiopulmonary bypass in dogs that sevoflurane posttreatment limits lung injury via Akt phosphorylation [27], suggesting a potential role for Akt signaling in the current model. But also other molecular pathways were described to mediate ROS formation, such as K ATP channel [14] or ERK 1/2 signaling [25].

### Sevoflurane posttreatment and inflammation

In addition to ROS formation, the release of pro-inflammatory cytokines and the transmigration of neutrophils into the alveolar compartments of the lungs are key events during the development of acute lung injury [30]. A series of pro-inflammatory cytokines have been described to promote VILI, such as IL-1β, MIP-1β, or MIP-2 [7,17,18,29,31]. Among them, the pro-inflammatory cytokine IL-1β appears to be of special interest. Its regulation and inhibition is required to finally achieve organ protection, since blockade of the IL-1 receptor has been demonstrated to inhibit neutrophil sequestration and edema formation in VILI [32]. In the current study, we clearly demonstrated that IL-1β and MIP-1β cytokine release as well as neutrophil transmigration were rapidly prevented by sevoflurane anesthesia. This finding is in line with recent work by ourselves and by others showing that sevoflurane treatment substantially reduced IL-1β release and neutrophil counts in the lung during mechanical ventilation in mice [18,19] or MIP-1β liberation from LPS challenged alveolar epithelial cells [40]. Interestingly, sevoflurane treatment had no effect on MIP-2 release, suggesting that MIP-2 signaling, in contrast to IL-1β, MIP-1β, or neutrophils, may not be involved in protection from VILI.

As discussed above, sevoflurane resolved histological signs of lung injury only when posttreatment was initiated early during ventilation. However, in contrast to these histological findings, but in line with the inhibition of ROS formation, lung inflammation was rapidly affected by sevoflurane application. Release of the pro-inflammatory cytokines IL-1β and MIP-1β and neutrophil transmigration were both prevented under sevoflurane posttreatment, irrespective of its onset or duration. Nonetheless, quite similar inhibitory effects of sevoflurane posttreatment on pro-inflammatory responses have been reported recently. In lipopolysaccharide challenged rats, for instance, sevoflurane posttreatment significantly reduced myeloperoxidase activity [41], tumor necrosis factor-α and IL-6 mRNA [42], or total cell counts and inflammatory mediators [43]. However, in all of these reports, rats received sevoflurane treatment as early as 2 h after the onset of LPS challenge for another 4 h; time dependent effects of sevoflurane treatment were not examined. Interestingly, a recent meta-analysis of patients who underwent cardiopulmonary bypass showed that serum levels of the pro-inflammatory cytokines IL-6 and IL-8 were significantly decreased in sevoflurane pre- and posttreated patients [20], supporting the notion that sevoflurane anesthesia inhibits the inflammatory response. In this regard our results add to these findings and show that sevoflurane posttreatment promptly prevents inflammation and oxidative responses, but inhibits lung injury in a time dependent manner during mechanical ventilation.

In the light of our results, extended investigations are needed to further unravel the molecular signaling pathways induced by sevoflurane posttreatment in protection from ventilator-associated lung injury.

## Conclusion

In the current study, we clearly showed that sevoflurane protects against VILI that is associated with inhibition of oxidative and inflammatory responses. When applied early during the insult, sevoflurane posttreatment exerts marked lung protection during mechanical ventilation. Moreover, inhibition of pro-inflammatory and oxidative responses is mediated by sevoflurane posttreatment, irrespective of its onset, highlighting the great organ-protective potential of sevoflurane aside from its safe sedative effects.
